# Therapeutic Low-Intensity Ultrasound for Peripheral Nerve Regeneration – A Schwann Cell Perspective

**DOI:** 10.3389/fncel.2021.812588

**Published:** 2022-01-05

**Authors:** Jenica Acheta, Shannon B. Z. Stephens, Sophie Belin, Yannick Poitelon

**Affiliations:** Department of Neuroscience and Experimental Therapeutics, Albany Medical College, Albany, NY, United States

**Keywords:** ultrasound, peripheral nerve regeneration, Schwann cells, LIU, LIPUS

## Abstract

Peripheral nerve injuries are common conditions that can arise from trauma (e.g., compression, severance) and can lead to neuropathic pain as well as motor and sensory deficits. Although much knowledge exists on the mechanisms of injury and nerve regeneration, treatments that ensure functional recovery following peripheral nerve injury are limited. Schwann cells, the supporting glial cells in peripheral nerves, orchestrate the response to nerve injury, by converting to a “repair” phenotype. However, nerve regeneration is often suboptimal in humans as the repair Schwann cells do not sustain their repair phenotype long enough to support the prolonged regeneration times required for successful nerve regrowth. Thus, numerous strategies are currently focused on promoting and extending the Schwann cells repair phenotype. Low-intensity ultrasound (LIU) is a non-destructive therapeutic approach which has been shown to facilitate peripheral nerve regeneration following nerve injury in rodents. Still, clinical trials in humans are scarce and limited to small population sizes. The benefit of LIU on nerve regeneration could possibly be mediated through the repair Schwann cells. In this review, we discuss the known and possible molecular mechanisms activated in response to LIU in repair Schwann cells to draw support and attention to LIU as a compelling regenerative treatment for peripheral nerve injury.

## Introduction

Peripheral nerve injuries refer to traumatic compression, cutting, or stretching of the peripheral nerve and cause a serious health problem that affects 2–3% of trauma patients annually (Noble et al., 1998; Taylor et al., 2008; Lad et al., 2010). Peripheral nerve injuries are classified according to their severity; grade I refers to reversible local conduction block, grades II and III refers to interruption of the axon and supporting structures, respectively, while grades IV and V refers to interruption of the nerve fascicle and all the nerve fibers, respectively (Sunderland, 1951). Because peripheral nervous system (PNS) axons can regrow, there is a frequent misbelief that neuronal damage can be repaired in the PNS without therapeutic strategies to support axon regeneration. Yet, in proximal injuries grade II and above, the axonal regrowth may occur but the long distance between the site of injury and the target organ greatly limits reinnervation. In addition, for grade IV/V injuries, when the nerve fascicle and/or fibers are separated, microsurgical repair is required to reconnect the nerve stump, but the regenerating axons fail to reinnervate their tissue targets (for review, see Menorca et al., 2013). Therefore, despite axon intrinsic regenerative potential, peripheral nerve regeneration following traumatic injury is often suboptimal, and generally results in life-long impairments, pain, and significant healthcare costs (Karsy et al., 2019; Bergmeister et al., 2020). Thus, additional therapies are explored to facilitate regeneration of all types of nerve injuries. In this review we will be focusing on the use of non-invasive therapeutic ultrasound in nerve regeneration.

An ultrasound is a sound wave above the human hearing threshold (above 20 kHz), which is commonly known for its clinical use in safe and non-invasive medical imaging. Despite being heavily used for diagnosis in imaging techniques, ultrasound can also generate mechanical energy. As the sound wave is being absorbed into biological tissue, it causes vibrations. The vibrations mediated by ultrasound are currently used in therapeutic settings through two main modalities: high intensity ultrasound and low-intensity ultrasound. High intensity ultrasound (>3 W/cm^2^) entails tissue molecular vibration, which converts into thermal generation (heat) and is used for the precise destruction of benign or malignant tissues. However, at low intensities (≤1 W/cm^2^), the thermal effect of sound waves is minimal or absent, thus causing no tissue damage, and is currently used to induce regenerative effects on biological tissues, to modulate nerve activity and to facilitate drug delivery.

Low-intensity ultrasound (LIU) is a non-thermogenic and non-destructive continuous wave with medium frequency ultrasound (1–3 MHz) and is delivered at low intensity. The frequency (i.e., the number of vibration cycles that occur in 1 s) of the LIU allows the sound wave to penetrate from 1 to 2 cm into biological tissues (1 MHz) up to 3–5 cm (3 MHz) (Takebe et al., 2014). LIU can be delivered through a pulse wave (i.e., in general ON for 20 μs and OFF for 80 μs). The pulsatile nature of ultrasound facilitates the emission of sound waves without heat generation (Grogan and Mount, 2021). Most studies thus far have used low intensity pulsed ultrasound (Tables 1, 2).

**TABLE 1:** Experimental parameters and outcomes of *in vivo* studies investigating the role of LIU on peripheral nerve after injury.

Study	Injury	Therapeutic regiment				Animal		Outcomes				
		*Parameters*	*Application*	*Duration*	*Length*	*Species*	*Sex*	*Timepoints*	*Morphological*	*Electrophysiogical*	*Functional recovery*	*Gene regulation*
Hong et al., 1988	Crush	500 mW/cm^2^, 1 Mhz	Every other day	1 min	n.d.	Rat	M		n.d.	Increased NCV and CMAP	n.d.	n.d.
Mourad et al., 2001	Crush	250 mW/cm^2^, 2.25 Mhz, continuous	Every other day	1 min	30 days	Rat	M	7, 14, 18, 22, 24, 26, 28, 30 dpi	n.d.	n.d.	Improved from 16 to 28 dpi	n.d.
Raso et al., 2005*	Crush	400 mW/cm^2^, 1 Mhz, 20% pulsed	Every day	10 min	10 days	Rat	M	7, 14, 21 dpi	Increased myelinated axon density at 21 dpi (STS)	n.d.	Improved at 14 and 21 dpi	n.d.
Chen et al., 2010	Crush	250 mW/cm^2^, 1 Mhz, continuous	Every other day	1 min	60 days	Rat	F	14, 30, 45, 60 dpi	Increased myelinated axon density from 30 to 60 dpi (IHC)	Increased NCV from 30 to 60 dpi	Improved from 30 to 60 dpi	Increased expression of NGF from 30 to 60 dpi (IHC)
Akhlaghi et al., 2012	Crush	500 mW/cm^2^, 1 Mhz, 20% pulsed	Every day	2 min	14 days	Mouse	n.d.	2, 4, 6,8, 10, 12, 14 dpi	n.d.	n.d.	Improved at 14 dpi	n.d.
Oliveira et al., 2012	Crush	400 mW/cm^2^, 1 Mhz, 20% pulsed	Every day	2 min	14 days	Rat	F	14 dpi	n.d.	n.d.	Improved at 14 dpi	n.d.
Jahromy et al., 2013	Crush	200 mW/cm^2^, 3.3Mhz, continuous	Every day	2 min	28 days	Rat	n.d.	4, 7, 14, 21, 28 dpi	n.d.	Increased CMAP at 7, 21 and 28 dpi	Increased at 28 dpi	Increased expression of CNTF at 14 and 28 dpi (qPCR)
Ni et al., 2017	Crush	200 mW/cm^2^, 1 Mhz, 20% pulsed	every day	1 min	30 days	Rat	M	7, 14, 21, 28 dpi	Increased myelin thickness from 21 to 28 dpi (EM)	Increased CMAP from 21 to 28 dpi	Improved from 14 to 28 dpi	n.d.
Ito et al., 2020 ^θ^	Crush	140 mW/cm2, 1 Mhz, 20% pulsed	5 days per week	5 min	21 days	Rat	M	3, 7, 21 dpi	Increased myelinated axon diameter and density at 21 dpi (STS & EM)	n.d.	not affected	Reduced expression of NT-3, GSK3β, TNF, IL-6, SEMA3A at 7 dpi (qPCR)
Wang et al., 2021 ^θ^	Crush	140 mW/cm^2^, 1 Mhz, 20% pulsed	every day for 2 weeks, then 5 days per week	5 min	30 days	Rat	M	3, 7, 14, 30 dpi	Increased axonal regrowth at 14 dpi and myelinated axon diameter, density and myelin thickness at 30 dpi (STS & EM)	n.d.	n.d.	Increased expression of BDNF at 14 dpi (qPCR)
Crisci and Ferreira, 2002	Cut	100 mW/cm^2^, 1.5 Mhz, 20% pulsed	Every day	20 min	12 days	Rat	M/F	12 dpi	Increased myelin thickness and myelinated axon density at 12 dpi (STS & EM)	n.d.	n.d.	n.d.
Chang and Hsu, 2004 ^§^	Cut(10 mm gap)+PLGA conduit	200 mW/cm^2^, 1Mhz, 20% pulsed	Every other day	5 min	14 days	Rat	M	45 dpi	Increased myelinated axon density at 45 dpi (IHC)	n.d.	n.d.	n.d.
Chang et al., 2005 ^§^	Cut(15 mm gap)+PLGA conduit	300 mW/cm^2^, 1 Mhz, 20% pulsed	Every other day	5 min	14 days	Rat	M	60 dpi	Increased myelinated axon density at 60 dpi (IHC)	n.d.	n.d.	n.d.
Park et al., 2010 ^†^	Cut(10 mm gap)+PLGA conduit	400 mW/cm^2^, 1 Mhz, 20% pulsed	Once a week	2 min	60 days	Rat	n.d.	30, 60 dpi	Increased myelin thickness and myelinated axon diameter at 30 and 60 dpi (STS & EM)	n.d.	n.d.	n.d.
Kim et al., 2013 ^†^	Cut(10 mm gap)+PLGA conduit	400 mW/cm^2^, 1 Mhz, 20% pulsed	Once a week	2 min	180 days	Rat	n.d.	30, 60, 120 dpi	Increased myelin thickness and myelinated axon diameter from 30 to 120 dpi (STS & EM)	Increased NCV from 90 to 120 dpi	n.d.	n.d.
Lv et al., 2015	Cut(10 mm gap)+PLGA conduit	300 mW/cm^2^, 1 Mhz, 20% pulsed	Every day	5 min	14 days	Rat	F	30, 90 dpi	n.d.	Increased NCV at 90 dpi	Improved at 30 and 90 dpi	n.d.
Jiang et al., 2016	Cut(10 mm autograft)	250 mW/cm^2^, 1 Mhz, 20% pulsed	Every other day	5 min	90 days	Rat	M	14, 30, 45, 60, 90 dpi	Increased myelin thickness, myelinated axon diameter and density at 90 dpi (STS & EM)	Increased CMAP at 90 dpi	Improved from 30 to 90 dpi	n.d.
Daeschler et al., 2018a	Cut	30 mW/cm^2^, 1.5 Mhz, 20% pulsed	Every day, or once a week	2 min	60 days	Rat	F	60 dpi	not affected (IHC)	not affected	not affected	n.d.

*The studies were categorized by type of injury (crush or transection) and in chronological order. List of the LIU parameters and measured outcomes of all analyzed studies including injury type, therapeutic regimen, animal and major outcomes on peripheral nerve morphology, electrophysiology, gene expression, and functional recovery following injury. §, †, θ these studies were done by the same lab. For this table, we use the PRISMA 2020 guidelines for systematic review (Page et al., 2021) and identified 19 reports. n.d., not determined. STS, semithin section. EM, electron microcopy. qPCR, quantitative PCR. IHC, immunohistochemistry. CMAP, compound muscle action potential. NCV, nerve conduction velocity. dpi, days post-injury.*

**TABLE 2:** Experimental parameters and outcomes of *in vitro* studies investigating the role of LIU on Schwann cells *in vitro*.

Study	Therapeutic regiment					Cells		Outcomes		
	*Transducer*	*Parameters*	*Application*	*Duration*	*Length*	*Cell culture*	*Number*	*Proliferation*	*Apoptosis*	*Gene regulation*
Zhang et al., 2009	Bottom of the plate	100 mW/cm^2^, 1 Mhz, 20% pulsed	Every day	5 min	14 days	Primary rat SC	3.000/cm^2^	Increased at day 4, 7 and 10	n.d.	Increased NT3 expression and decreased BDNF expression at day 14 (RT-PCR)
Tsuang et al., 2011	Immersed	300 mW/cm^2^, 1 Mhz, 20% pulsed	Once	2 min	2 days	Primary rat SC	2.000/cm^2^	Increased at day 2	Decreased at day 1	n.d.
Yue et al., 2016	Bottom of the plate	20 mW/cm^2^, 1 Mhz, 20% pulsed	Every day	10 min	7 days	Rat SC RSC96	60.000/cm^2^	n.d.	n.d.	Increased ErbB3, NRG1, EGR2 and MBP expression at day 1, 4 and 7 (qPCR)
Ren et al., 2018	Bottom of the plate	27.500 mW/cm2^†^	Every day	10 min	5 days	primary rat SC	2.000/cm2	Increased at day 5	n.d.	Increased FDF, NGF, BDNF, GDNF, p-GSK-3β, β-catenin expression at day 5 (WB)

*Listed are the key elements of all analyzed studies including therapeutic regimen, cell type and number and major outcomes on Schwann cell proliferation, apoptosis, and gene expression. †, Ren et al. (2018) reported using an intensity of 27.5 W/cm^2^, which is considered high intensity ultrasound. For this table, we use the PRISMA 2020 guidelines for systematic review (Page et al., 2021) and identified 4 reports. SC, Schwann cell. n.d., not determined. qPCR, quantitative PCR. RT-PCR, reverse transcriptase PCR. WB, western blot.*

Low-intensity ultrasound was approved by the US FDA about 27 years ago for fracture repair. Since then, a growing amount of literature demonstrates that LIU non-invasive physical stimulus can stimulate or inhibit physiological processes and facilitate drug delivery. More specifically, LIU can accelerate soft-tissue regeneration (e.g., muscles, tendons, ligaments) (Ikai et al., 2008; Jeremias Junior et al., 2011; Ren et al., 2013), inhibits inflammatory responses (Nakao et al., 2014; Zhao et al., 2017), and modulates neuronal activity (Iwashina et al., 2006; Su et al., 2017; Zou et al., 2021). For a more comprehensive review the therapeutic applications of LIU, see (Xin et al., 2016; Jiang et al., 2019; de Lucas et al., 2020; Uddin et al., 2021). While the application of LIU could also be of great benefit to nerve repair by promoting neuromodulation, neuronal regrowth and neuromuscular rehabilitation, the clinical efficacy of LIU in neuromuscular trauma and neurodegenerative diseases is understudied. Thus, the utilization of LIU in nerve regenerative medicine is still limited. More pre-clinical and clinical studies are necessary to evaluate LIU as a suitable clinical tool in nerve repair. We review here the effect of LIU on peripheral nerve regeneration in rodent pre-clinical studies and more specifically the effect of LIU on Schwann cells, the supporting glial cells of the PNS, known for their capacity to reprogram into repair cells to promote nerve repair.

## Effect of Low-Intensity Ultrasound on Peripheral Nerve Regeneration

Given the limitations of peripheral nerves to self-heal in humans, therapeutic approaches to promote nerve regeneration must be developed. Over the last 20 years, numerous groups have investigated the therapeutic effect of LIU on peripheral nerve injury to facilitate regeneration and improve function both in pre-clinical (Table 1) and clinical settings. For a recent meta-analysis of functional outcomes in pre-clinical studies, see (Daeschler et al., 2018b). For a recent meta-analysis of clinical studies, see (Haffey et al., 2020). Our review will point out how the research on the effect of LIU application in nerve repair is considerably scattered between type of injury model used, the LIU parameters applied, the experimental paradigm utilized, and the choice of the outcomes measured. Despite the divergent therapeutic regiment used in pre-clinical studies, a few LIU constants were identified for their therapeutic effect on peripheral nerve regeneration (i.e., morphological and functional improvement). First, LIU intensity needs to be between 200 and 500 mW/cm^2^. At lower intensity (≤100 mW/cm^2^), the beneficial effect of LIU is not observable (Daeschler et al., 2018a; Ito et al., 2020), while at higher intensity (≥1 W/cm^2^) the beneficial effect is reduced or absent (Hong et al., 1988; Mourad et al., 2001; Akhlaghi et al., 2012; Jiang et al., 2016). Second, to improve nerve regeneration, LIU should be applied repetitively, either every day or every other day and for a short period of time (between 1 and 10 min per application). Third, none of the studies have reported negative side effects resulting from LIU, such as limiting or impairing peripheral nerve regeneration (Table 1). However, because it has not been investigated yet, it is unclear if a longer and/or more repetitive application of LIU will be beneficial or become detrimental for nerve regeneration.

In summary, multiple advances made from pre-clinical studies lead to the current consensus that LIU application promotes peripheral nerve regeneration after peripheral nerve injuries (Hong et al., 1988; Mourad et al., 2001; Crisci and Ferreira, 2002; Chang and Hsu, 2004; Chang et al., 2005; Raso et al., 2005; Chen et al., 2010; Park et al., 2010; Akhlaghi et al., 2012; Oliveira et al., 2012; Jahromy et al., 2013; Kim et al., 2013; Lv et al., 2015; Jiang et al., 2016; Ni et al., 2017; Ito et al., 2020; Wang et al., 2021). More precisely, it was shown that LIU could: (i) increase the number, diameter, or the myelination of axon distal to the lesion site; (ii) improve nerve conduction velocities (NCV) and compound muscle action potentials (CMAP); and (iii) enhance functional recovery after nerve injury (Table 1) (for review, see Peng et al., 2020). In addition, a few studies have shown that application of LIU on injured nerves is sufficient to alter gene regulation of neurotrophic factors, cytokines, or promyelinating genes during peripheral nerve regeneration (Chen et al., 2010; Jahromy et al., 2013; Ito et al., 2020; Wang et al., 2021). However, despite these encouraging studies, LIU application on peripheral nerve injury is not considered, possibly because the precise cellular and molecular mechanisms supporting the therapeutic effect of LIU during peripheral nerve regeneration are still not understood.

## Effect of Low-Intensity Ultrasound on Schwann Cells

During peripheral nerve regeneration, repair Schwann cells fulfill a sequence of supportive functions for injured axons to survive, regenerate and reinnervate their tissue target. These include the expression of trophic factors to prevent neuronal death, the expression of cytokines to recruit macrophages, the autophagy of myelin debris, the formation of regeneration tracks to guide axonal regrowth, and eventually the remyelination of axons. Therefore, LIU regenerative effects could be mediated through repair Schwann cells and their numerous pro-regenerative properties that are essential to the nerve repair. While all morphological and electrophysiological outcomes observed *in vivo* in peripheral nerves after injury suggest that LIU acts on repair Schwann cells (Table 1), specific assessments of repair Schwann cell function and their differentiation into myelinating or non-myelinating Schwann cells remains unstudied. In addition, only a few groups have looked at the effect of LIU on primary Schwann cells *in vitro* (Zhang et al., 2009; Tsuang et al., 2011; Yue et al., 2016; Ren et al., 2018; Table 2). A consistent effect of LIU on Schwann cells *in vitro* (observed in 3 out of 4 studies) is an increase of Schwann cell proliferation following the first days after LIU application (Zhang et al., 2009; Tsuang et al., 2011; Ren et al., 2018). Ren et al. (2018) proposed that, the increased Schwann cell proliferation was mediated by enhancing cyclin D1 expression, similar to what was found with LIU in other cell types (i.e., mesenchymal stem cells and chondrocytes) (Takeuchi et al., 2008; Ling et al., 2017; Xie et al., 2019). Yet, while LIU may activate mitogenic signals in Schwann cells, the increase in repair Schwann cell proliferation following LIU application has yet to be studied *in vivo*. In addition, it is now known that proliferation is not critical for peripheral nerve regeneration in cyclin D1-null mice (Yang et al., 2008), contrasting with Ren et al. (2018) hypothesis and implying that further studies are necessary to identify the molecular mechanisms responsible for the LIU-mediated nerve repair.

## Mechanisms of Action of Low-Intensity Ultrasound on Schwann Cells

### Neurotrophic Factors

Following LIU, the observed increase in the number of myelinated axons, as well as the improvement in CMAP (Jahromy et al., 2013; Jiang et al., 2016; Ni et al., 2017), suggests that LIU improves the regrowth of axons. One hypothesis is that the regrowth of axons is mediated through the secretion of neurotrophic factors (i.e., NT-3, FGF, NGF, BDNF, and GDNF) by Schwann cells. Neurotrophic factors have been shown to promote neuroprotection, axonal regrowth and even myelinogenesis following peripheral nerve injury (for review, see Li et al., 2020). Six studies, using various therapeutic regiment, have looked at the effect of LIU on neurotrophic factor expression in Schwann cells (Zhang et al., 2009; Chen et al., 2010; Jahromy et al., 2013; Ren et al., 2018; Ito et al., 2020; Wang et al., 2021). However, it remains unclear how parameter changes in LIU show contrasting effects on neurotrophic factor secretion and how neurotrophic factor secretion will be modulated by LIU after more severe injury (nerve cut). Thus, further comprehensive *in vitro* and *in vivo* studies are needed to clarify how different LIU therapeutic regimens regulate Schwann cell neurotrophic factor expression and secretion.

### Pro-inflammatory Cytokines

Within a few hours following injury, repair Schwann cells release pro-inflammatory cytokines and interleukins (e.g., TNFα, IL-6) that promote the massive recruitment of macrophages distal to the injury. Macrophages, in conjunction with repair Schwann cells, clean the myelin debris and help restructure the extracellular matrix. In addition, cytokines (e.g., IL-6) have direct pro-regenerative roles; promoting axonal (Hirota et al., 1996) and blood vessel (Cattin et al., 2015) regrowth at the axonal stump. One group showed that 7 days after nerve crush injury in rats, LIU suppressed the expression of inflammatory cytokines TNFα and IL-6, suggesting that LIU attenuate the pro-inflammatory response during peripheral nerve regeneration (Ito et al., 2020). While the positive modulation of the inflammatory response could improve peripheral nerve regeneration (for review, see Dubový et al., 2013), more comprehensive studies are necessary to decipher how the effect of LIU on the inflammatory responses participates in the LIU-mediated functional reinnervation improvement.

### Schwann Cell Redifferentiation and Remyelination

Low-intensity ultrasound in peripheral nerves following injury is consistently found to promote myelin thickening and increase NCV (Crisci and Ferreira, 2002; Chen et al., 2010; Park et al., 2010; Kim et al., 2013; Lv et al., 2015; Jiang et al., 2016; Ni et al., 2017). These observations suggest that application of LIU promotes repair Schwann cell redifferentiation into myelinating Schwann cells and/or remyelination. Yue et al. showed that *in vitro* application of LIU on Schwann cells increases the expression of proteins involved in Schwann cells myelination: ErbB3; a receptor of juxtracrine and autocrine promyelinating neuregulin 1, EGR2; a transcriptional regulator for Schwann cell myelination, and MBP; a major myelin protein (Yue et al., 2016). However, considering this study was performed on Schwann cells in culture in non-myelinating conditions, the promyelinating effect of LIU on myelin wrapping remains to be demonstrated either *in vivo* or in Schwann cell/neuron myelinating co-culture.

## Mechanisms of Action of Low-Intensity Ultrasound Independent of Schwann Cells

While most data support that the therapeutic effect of LIU on peripheral nerve regeneration is mediated by a direct effect on repair Schwann cells, an alternative hypothesis is that the improvement following LIU application is mediated directly through axons. Ventre et al. (2018, 2021) demonstrated *in vitro* that application of LIU on dorsal root ganglia neurons increased neurite outgrowth by two-fold compared to untreated controls, possibly by activating the Netrin-1/DCC pathway (Wen et al., 2021). In addition, it was suggested that LIU promoted axonal regrowth through the decrease of axonal semaphorin 3A expression, an inhibitor of axonal regeneration, and the decrease of GSK-3β, a potential inhibitor of axonal regrowth (Ito et al., 2020). However, because of conflicting reports on the role of GSK-3β signaling as either a beneficial or detrimental pathway for axon regeneration (Ogata et al., 2004; Zhou et al., 2004; Dill et al., 2008; Kim and Snider, 2011), the modulation of GSK-3β signaling by LIU and it contribution to nerve repair remains unclear. Following peripheral nerve injury, LIU could modulate myeloid cells (innate immune responses) (Xu et al., 2021) or vascular endothelial cells (angiogenesis) (de Lucas et al., 2020). The effect of LIU on these cells during peripheral nerve regeneration context have not been studied.

## New Avenues of Research on Identifying the Low-Intensity Ultrasound-Mediated Sensing Mechanisms in Schwann Cells

A major gap of knowledge in the current field is how LIU is sensed by Schwann cells. In this review, we analyzed the known effects of LIU in other cellular systems as they may be translated to Schwann cells. LIU application was initially implicated for the treatment of bone fracture and arthritis, thus most of the known LIU-sensitive pathways were established *in vitro* from articular joint cell types. In cartilage and synovial cells, application of LIU increases the expression of extracellular matrix (ECM) components which in turns activates ECM-bound receptor responses such as the integrin/FAK/PI3K/AKT pathway (Choi et al., 2007; Takeuchi et al., 2008; Naito et al., 2010; Whitney et al., 2012; Cheng et al., 2014; Sato et al., 2014; Xia et al., 2015; Zhang et al., 2016; Ding et al., 2020). LIU was found to activate similar pathways in other cell types, such as fibroblasts, keratinocytes and mesenchymal stem cells (Bohari et al., 2012; Leng et al., 2018; Chen et al., 2019; Hormozi-Moghaddam et al., 2021) which strongly suggest that the integrin/FAK/PI3K/AKT pathway activation by LIU may not be limited to certain cell-type. Macromolecules of the ECM and basal lamina, such as collagen, laminin, and fibronectin, constitute the microenvironment of Schwann cells. Schwann cells harbor receptors for the ECM, including Integrins, GPR126, and dystroglycan. The ECM interact with their receptors which activates cascades of phosphorylation in part through RhoGTPases, Focal Adhesion Kinase (FAK) and Integrin-Linked Kinase (ILK). These receptors and kinases contribute to the transmission of mechanical signals from the ECM to the nucleus (for review, see Martino et al., 2018), and are critical for Schwann cell development and myelination (for review, see Monk et al., 2015; Feltri et al., 2016; Wilson et al., 2021). Importantly, those same receptors and kinases have also been shown to be required for peripheral nerve regeneration following injury (Werner et al., 2000; Akassoglou et al., 2002; Chen and Strickland, 2003; Van der Zee et al., 2008; Pereira et al., 2009; Chen et al., 2015; Mogha et al., 2016; Atherton et al., 2017; Zainul et al., 2018). In addition, recent studies have shown that stimuli from the ECM can lead to the reorganization of the actin cytoskeleton, which induces the activation of transcriptional coactivators YAP/TAZ (Dupont et al., 2011; Zhao et al., 2012; Aragona et al., 2013; Totaro et al., 2017), and Rho GTPase (Aragona et al., 2013; Reginensi et al., 2013). Two studies have shown that LIU application leads to the activation of YAP/TAZ in endothelial cells and retinal ganglion cells (Xu et al., 2018; Zhou et al., 2018). This suggests that LIU application could modulate diverse pathways in Schwann cells through changes in ECM composition, architecture, and the alteration of the interactions between the ECM and ECM-bound receptors. Yet, it is likely that in response to LIU, mechanotransduction would initiate multiple signaling pathways at once, and these pathways can have significant crosstalk and overlap, making it difficult to associate the observed improvement in peripheral nerve regeneration to one specific pathway.

## Conclusion

Over the last 20 years, both pre-clinical and clinical studies have attempted to characterize the effect of LIU on peripheral nerve regeneration. There are many compelling evidence that application of LIU increase the number, diameter, or the myelination of axon distal to the lesion site, improve functional outcomes and globally enhance peripheral nerve regeneration after nerve injury. Yet, there is still a need for studies with comprehensive mechanistic results to understand how LIU sound waves affect the regenerative processes following peripheral nerve injury. While it is likely that the effect of LIU is mediated through repair Schwann cells, which are the central hub for peripheral nerve regeneration, the demonstration is still lacking. It is still unclear how LIU affects ECM composition, ECM-mediated signaling pathways; which could mediate repair Schwann cells’ fate during peripheral nerve regeneration. In addition, independently from Schwann cells, the effect of LIU on immune and vascular cells, known to contribute to nerve repair is currently unknown. Future studies should also evaluate the effect of LIU on neuropathic pain and investigate LIU in sensory nerves, as all functional studies have focused on motor outcomes so far. There are a few reports indicating that peripheral nerve regeneration may be sexually dimorphic as axonal regrowth seems to be more efficient in males (Stenberg and Dahlin, 2014), while remyelination post-injury is more efficient in females (Kovacic et al., 2004; Tong et al., 2015). Thus, future research will need to carefully evaluate how peripheral nerve regeneration mediated by LIU may differ between sexes. Further understanding of LIU’s *modus operandi* on peripheral nerve injury would likely support further clinical trial assessing the therapeutic effect of LIU during peripheral nerve regeneration in humans.

## Author Contributions

JA, SB, and YP wrote the manuscript. SB, SS, and YP revised the manuscript. YP created the tables. All authors approved the final version of the manuscript for publication.

## Conflict of Interest

The authors declare that the research was conducted in the absence of any commercial or financial relationships that could be construed as a potential conflict of interest.

## Publisher’s Note

All claims expressed in this article are solely those of the authors and do not necessarily represent those of their affiliated organizations, or those of the publisher, the editors and the reviewers. Any product that may be evaluated in this article, or claim that may be made by its manufacturer, is not guaranteed or endorsed by the publisher.
